# Differential microRNA profiles in elderly males with seborrheic dermatitis

**DOI:** 10.1038/s41598-022-24383-3

**Published:** 2022-12-08

**Authors:** Hyejun Kim, Jae Won Yun, Gayun Baek, Sungchul Kim, Mihn-Sook Jue

**Affiliations:** 1 School of Biological Sciences, Seoul National University, Seoul, 08826 Korea; 2 Center for RNA Research, Institute for Basic Science, Gwanak-ro 1, Gwanak-gu, Seoul, 08826 Korea; 3 Veterans Medical Research Institute, Veterans Health Service Medical Center, Seoul, 05368 Korea; 4 Department of Dermatology, Veterans Health Service Medical Center, Seoul, 05368 Korea; 5 Department of Dermatology, Hanyang University Hospital, 222-1 Wangsimniro, Seongdong-gu, Seoul, 04763 Korea

**Keywords:** Non-coding RNAs, RNA metabolism

## Abstract

Seborrheic dermatitis (SD) is one of the most common skin diseases characterized by inflammatory symptoms and cell proliferation, which has increased incidence in patients older than 50 years. Although the roles of microRNAs (miRNAs) have been investigated in several diseases, miRNA profiles of patients with SD remain unknown. This study aimed to identify differentially expressed miRNAs (DEMs) in lesions of elderly male patients with SD. We used a microarray-based approach to identify DEMs in lesions compared to those in non-lesions of patients with SD. Furthermore, Gene Ontology and pathway enrichment analysis were performed using bioinformatics tools to elucidate the functional significance of the target mRNAs of DEMs in lesions of patients with SD. Expression levels of two miRNAs—hsa-miR-6831-5p and hsa-miR-7107-5p—were downregulated, whereas those of six miRNAs—hsa-miR-20a-5p, hsa-miR-191-5p, hsa-miR-127-3p, hsa-miR-106b-5p, hsa-miR-342-3p, and hsa-miR-6824-5p—were upregulated. Functions of the SD-related miRNAs were predicted to be significantly associated with typical dermatological pathogenesis, such as cell proliferation, cell cycle, apoptosis, and immune regulation. In summary, SD alters the miRNA profile, and target mRNAs of the DEMs are related to immune responses and cell proliferation, which are the two main processes in SD pathogenesis.

## Introduction

Seborrheic dermatitis (SD) is a common chronic inflammatory skin disease worldwide^1,2^. Clinically, SD is characterized by erythematous greasy patches with yellowish scales on seborrheic areas such as the scalp, face, upper chest, and retroauricular area and can affect people of various ages and races. Although SD is neither contagious nor fatal, its clinical characteristics, including skin lesions in the exposed area, dandruff, and chronic and recurring course, can have a considerable negative effect on the patient’s quality of life and self-esteem. The incidence of SD in males is approximately threefold higher than that in females, and its prevalence increases in patients aged > 50 years^3^. Previous studies have also shown that the prevalence of SD in the elderly is consistently higher than that in the general population^4,5^ and has been reported to be as high as 31%^6^.

Several studies have been performed to determine the etiology of SD, with most studies focusing on the role of *Malassezia* in the disease pathogenesis^7–10^. Other recent studies have emphasized the role of individual susceptibility to abnormal inflammatory responses, alterations in the epidermis, and underlying genetics^11–14^. However, the pathology and molecular features of SD remain unclear.

MicroRNAs (miRNAs) are small non-coding RNAs, approximately 22 nucleotides in length, expressed from miRNA encoding regions in the genome. miRNAs are loaded onto effector Argonaute proteins and generally bind to the 3′-untranslated region of their target mRNAs through seed (a 2–7-nucleotide region in a miRNA)-dependent hybridization, eventually resulting in either degradation or translational inhibition of the target mRNAs^15,16^. miRNAs have been implicated as cellular regulatory factors in several cellular and developmental processes, such as inflammation, cell differentiation, cell death, and proliferation^17–19^. From a clinical perspective, miRNAs have been intensively studied in various human diseases such as cancers, metabolic disorders, infectious diseases, and immunological disorders as biomarkers and therapeutic targets^20^. The pathologies modulated by miRNAs have been examined in dermatologic diseases such as psoriasis^21–23^, atopic dermatitis^24^, and allergic contact dermatitis^25,26^. However, little is known about the roles of miRNAs in SD.

This study investigated differentially expressed miRNA (DEM) profiles and their potential targets in SD. To identify miRNAs with upregulated and downregulated expression in SD, we conducted microarray-based miRNA profiling using biopsy samples obtained from elderly male patients with SD, followed by a detailed bioinformatic analysis. To determine the biological roles of miRNAs, we analyzed the potential target mRNAs of DEMs using computational prediction. We also predicted the mechanism and causality through Gene Ontology (GO) and pathway analyses.

## Results

### Quality evaluation of the microarray analysis

**Figure 1:**
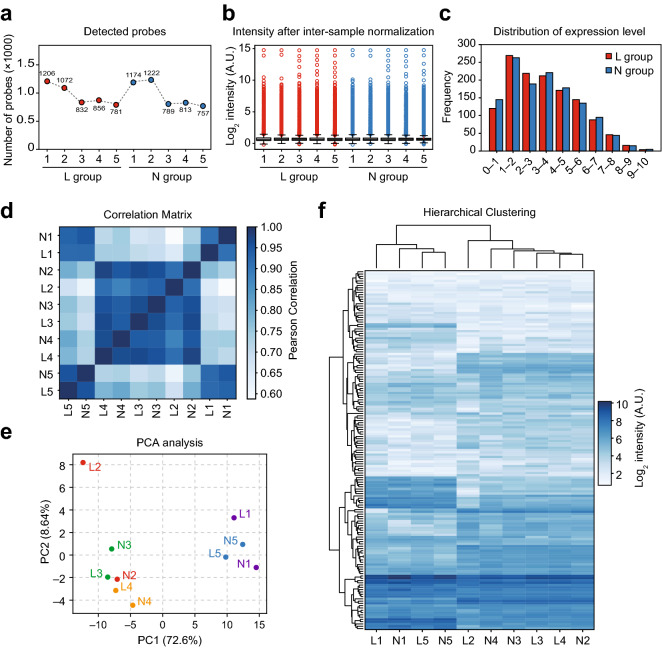
Quality evaluation of the miRNA microarray data. (**a**) Number of miRNA probes detected in each sample group. (**b**) Density plot showing the signal distribution of each sample group. (**c**) Box plot of the percentile values for the normalized expression levels of each sample group. (**d**) Correlation matrix for the degree of similarity, (**e**) principal component analysis (PCA), and (**f**) hierarchical clustering among the sample groups.

First, we conducted microarray analyses to determine the expression profiles of mature miRNAs that were differentially expressed in each lesion (L) compared to the corresponding non-lesion (N) biopsy samples obtained from five patients with SD. After data preprocessing, the quality of miRNA detection analysis with microarray for each sample was verified by preparing the number of detected probes (Fig. 1a) to analyze the distribution of the normalized signals (Fig. 1b) and distribution of miRNA expression levels (Fig. 1c). Furthermore, intersample correlation analysis (Fig. 1d, Supplementary Fig. 1), principal component analysis (PCA) (Fig. 1e) and hierarchical clustering (Fig. 1f) were conducted to analyze the overall structure of the microarray data and similarity among samples. Although samples originating from five different individuals were expected to be categorized into two main L and N groups, samples from the same person were more correlated than samples from the same group (L or N). Since microarray analyses of patients 1 and 5 (L1, N1, L5, and N5) were separately conducted in a different batch from those of patients 2, 3, and 4 (L2, N2, L3, N3, L4, and N4), a distinct correlation of the same batch was observed (Fig. 1d–f, Supplementary Fig. 1). Therefore, samples from the same individuals were paired and compared in further investigations to minimize the batch effect and biased difference among individuals.

### DEMs between lesion and non-lesion in SD

**Figure 2:**
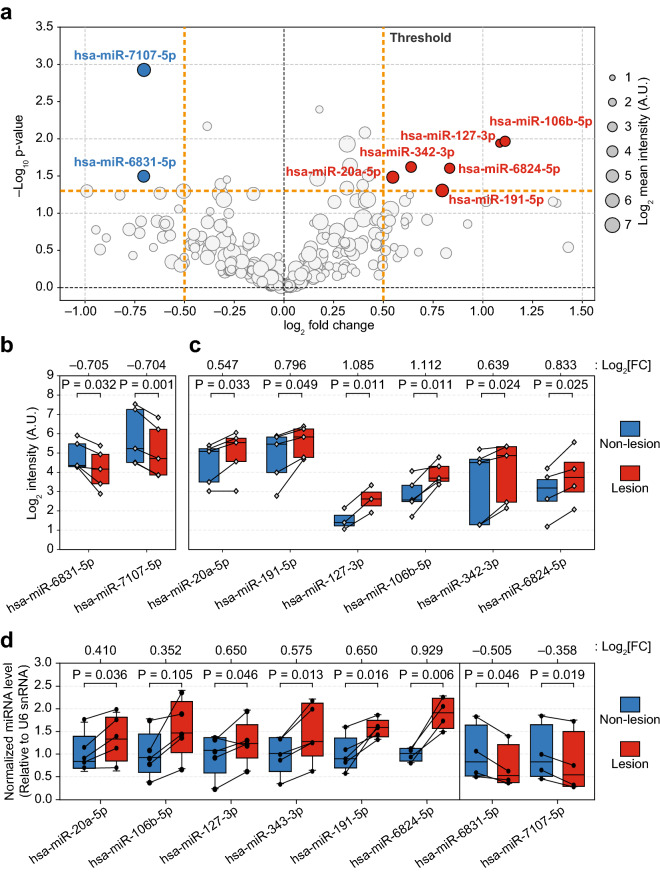
Differentially expressed miRNAs (DEMs) between the lesion and non-lesion samples from patients with SD. (**a**) Volcano plot of − log_10_ [*P*-value] against log_2_ [fold-change, FC], showing the difference in miRNA expression between tissues from the lesion (*n* ≥ 3) and non-lesion (*n* ≥ 3). Levels of miRNAs marked as red were upregulated in the lesion tissue, and those marked as blue were downregulated. The horizontal dashed line (orange bold) indicates the threshold for statistical significance (paired *t*-test, *P* < 0.05), and the vertical dashed lines (orange bold) indicate the threshold FC (*log*_*2*_*|FC|* > 0.5). The size of the bubbles shows their relative intensities in the microarray. (**b**,**c**) Box plots represent DEMs in the microarray data. Each diamond symbol indicates each sample. Paired samples are connected to each other (see Supplementary Table S1 online). (**b**) Expression levels of hsa-miR-6831-5p (*n* = 5) and hsa-miR-7107-5p (*n* = 5). (**c**) Expression levels of hsa-miR-20a-5p (*n* = 5), hsa-miR-191-5p (*n* = 5), hsa-miR-127-3p (*n* = 3), hsa-miR-106b-5p (*n* = 5), hsa-miR-342-3p (*n* = 5), and hsa-miR-6824-5p (*n* = 4). (**d**) The qRT-PCR verification of the mature miRNA levels. The relative intensity was measured based on the expression levels of the U6 snRNA, an internal control, and further normalized with each L group miRNA level by the mean value of N group miRNA levels. Each dot indicates a sample. Paired samples are connected to each other. *P*-values (two-tailed Paired *t*-test) and fold changes (*log*_*2*_*|FC|*) are indicated in each panel.

To discover DEMs in SD lesions compared to non-lesions, we performed statistical and fold-change (FC) analyses based on the miRNA profiles of the L and N groups. Eight DEMs exhibited significant alterations with *P*-values < 0.05 and *log*_*2*_*|FC|* > 0.5 (Fig. 2a; Supplementary Table S1). Expression levels of two miRNAs, hsa-miR-6831-5p and hsa-miR-7107-5p, were downregulated (Fig. 2b), whereas those of six miRNAs, hsa-miR-20a-5p, hsa-miR-191-5p, hsa-miR-127-3p, hsa-miR-106b-5p, hsa-miR-342-3p, and hsa-miR-6824-5p were upregulated (Fig. 2c). Notably, both hsa-miR-20a-5p and hsa-miR-106b-5p presented the same seed sequence (5′-AAAGUGC-3′). To validate the reliability of our finding, quantitative real-time polymerase chain reaction (qRT-PCR) was performed for these eight miRNAs using RNA samples that were used in microarray analyses. The expression of up- and downregulated miRNAs between L and N samples were comparable to the results obtained by microarray analysis (Fig. 2d). Therefore, we selected these eight miRNAs as DEMs in elderly male patients with SD for the subsequent investigations.

### Potential target mRNAs of DEMs

Next, we evaluated the potential target mRNAs of the DEMs by experimental or computational prediction of the miRNA–mRNA interactions. To predict these targets, we used the open-source TargetScan algorithm (release version 8.0, September 2021), considering only miRNA–mRNA interactions with high confidence. A total of 893 mRNAs of the eight DEMs were identified as a non-redundant target set (Table 1; Supplementary Table S2). The level of mRNA targets tends to be downregulated by corresponding miRNAs^15,16^. Therefore, out of the 893 mRNA targets, 243 mRNA targets of hsa-miR-6831-5p and hsa-miR-7107-5p, and 650 mRNA targets of hsa-miR-20a-5p, hsa-miR-191-5p, hsa-miR-127-3p, hsa-miR-106b-5p, hsa-miR-342-3p, and hsa-miR-6824-5p that were predicted to be potentially upregulated and downregulated, respectively, in the L group compared to the N group (Supplementary Table S2) were selected for further GO and functional enrichment analyses.

**Table 1:** Differentially expressed miRNAs (DEMs) with their seed sequences and the number of target mRNAs.

miRNA	Seed sequence (5′-to-3′)	Number of targets
hsa-miR-20a-5p/hsa-miR-106b-5p	AAAGUGC	223
hsa-miR-6824-5p	UAGGGGA	166
hsa-miR-6831-5p	AGGUAGA	137
hsa-miR-127-3p	CGGAUCC	108
hsa-miR-7107-5p	CGGCCUG	106
hsa-miR-342-3p	CUCACAC	95
hsa-miR-191-5p	AACGGAA	58

### Functional analysis of target mRNAs potentially related to SD pathology

**Figure 3:**
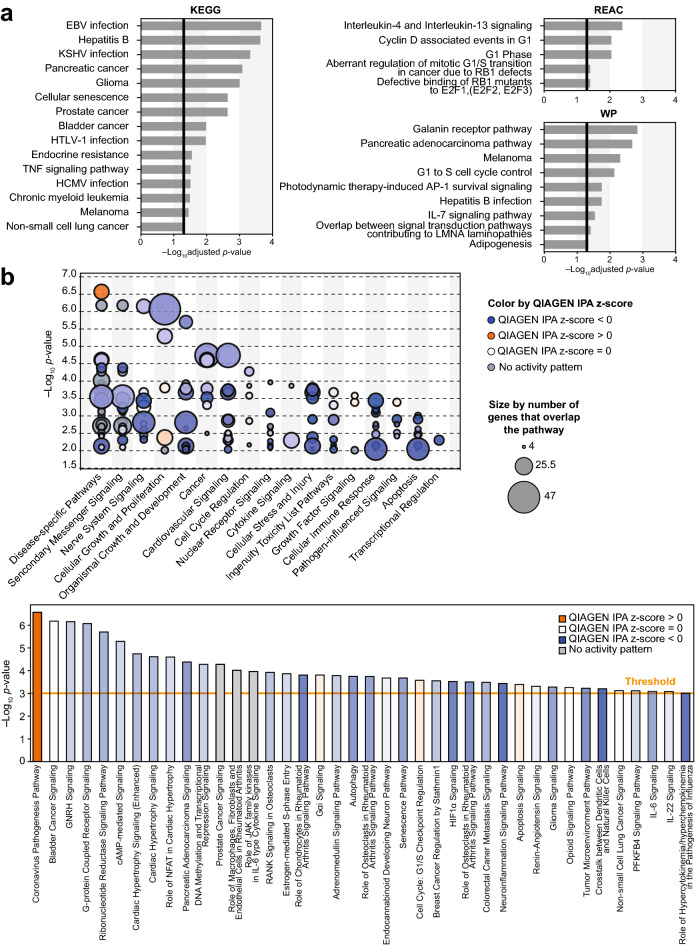
Functional enrichment analysis of potential target mRNAs of DEMs in patients with SD. (**a**) GO analysis of target mRNAs using g:GOSt of gProfiler. Three data sources were used: Kyoto Encyclopedia of Genes and Genomes (KEGG), REACTOME (REAC), and WikiPathways (WP). The vertical black lines indicate the threshold for the adjusted *P*-value (*Q* < 0.05) (See Supplementary Table S3). (**b**) Bubble (top) and bar (bottom) charts based on Canonical Pathway scores arranged by the IPA categories using SD-regulated target mRNAs of DEMs. Orange and blue-colored bubbles or bars: pathway activation or predicted inhibition, respectively, based on QIAGEN IPA z-score, gray bars: pathways for which no prediction could be made because of insufficient evidence in the Knowledge Base for confident activity predictions across datasets, white: pathways with QIAGEN IPA z-scores at or very close to 0. The bubble size corresponds to the number of overlapping genes in each pathway (shown at the extreme right). The *P*-value was calculated using right-tailed Fisher's exact test, such that taller bars equate to increased significance. The threshold of − log(*P*-value) in the bar chart is 3.

Functional enrichment analysis of the DEMs was conducted using the Kyoto Encyclopedia of Genes and Genomes (KEGG), WikiPathways (WP), and REACTOME (Supplementary Table S3). Particularly, GO terms related to the regulation of immune responses against viral infections, cancer, regulation of cell cycle, and interleukin signaling were considerably over-represented (Fig. 3a). As SD is reportedly highly related to *Malassezia* yeasts^10,27,28^, and *Malassezia* species can induce skin immune responses^11–14^, enriched terms for several immune-related genes may be notable.

**Figure 4:**
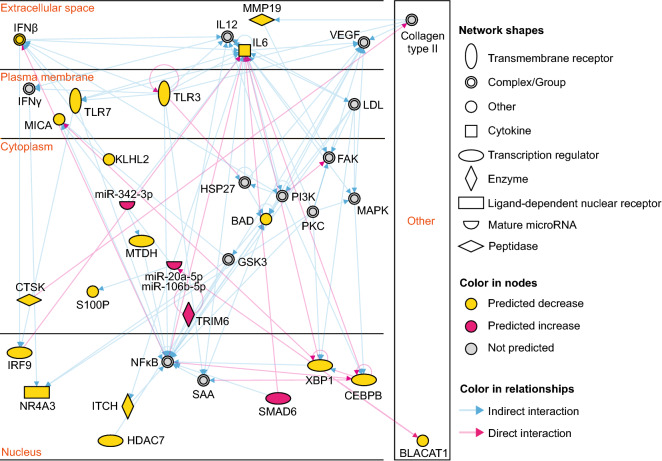
Target pathways related to immune response underlying SD. Immune regulation-related example of Ingenuity Pathway Analysis networks (score: 33) generated by analyzing DEMs and their target mRNAs (transcripts in Supplementary Table S2).

Pathway analysis was performed using the QIAGEN Ingenuity Pathway Analysis (IPA) tool (version released in September 2021). Similar to the results of GO analysis, immune-related and cell proliferation/death-related pathways, such as cytokine signaling, immune cell crosstalk and activation, cell cycle regulation, and pathogenesis, were substantially enriched (Fig. 3b, Supplementary Fig. 2). For instance, in the highest-scored networks, we detected several genes related to immune responses, such as those encoding Toll-like receptors, interleukins, interferons, and interferon regulatory factors (Fig. 4). Thus, the mRNA targetome of the DEMs in SD reflects gene expression regulation related to immune responses and cell proliferation, which are two main processes in SD pathogenesis.

## Discussion

We investigated the pattern of altered miRNA expression in the skin tissues of lesions compared to non-lesions in five patients with SD. The DEM profiles were analyzed through a series of bioinformatics analyses. The expression levels of hsa-miR-6831-5p and hsa-miR-7107-5p were downregulated, while those of hsa-miR-20a-5p, hsa-miR-191-5p, hsa-miR-127-3p, hsa-miR106b-5p, hsa-miR-342-3p, and hsa-miR-6824-5p were upregulated. Furthermore, we predicted the potential target mRNAs of these DEMs and characterized the functional roles of the predicted targets correlated with SD pathology.

SD is a refractory skin disorder; its development depends on individual susceptibility, which is influenced by various intrinsic and extrinsic factors, such as abnormal sebaceous gland activity, *Malassezia* colonization, epidermal barrier integrity, immune responses, neurogenic/nutritional factors, emotional/extrinsic stresses, and genetic backgrounds^29,30^. Therefore, our DEM data provide valuable insight into the molecular and cellular mechanisms underlying SD pathogenesis concerning the role of small non-coding RNA such as miRNA, which has not been previously explored in relation to this skin disease.

The substantially regulated miRNAs may not be specific to SD but may also be commonly represented in other inflammatory skin diseases, such as psoriasis and atopic dermatitis. For instance, SD shares several clinical characteristics with other chronic inflammatory skin disorders, particularly psoriasis and atopic dermatitis^29^. In this regard, it is noteworthy that hsa-miR-20a/106b-5p seed group miRNAs, which were considerably upregulated in SD lesions (Fig. 2), were also upregulated in psoriasis^21,22^ and urine from children with atopic dermatitis^24^. The functional features of the predicted targetomes were over-represented in cell proliferation, cell death, and immune responses (Fig. 3, Supplementary Fig. 2), indicating that in SD, miRNA potentially regulates pathological processes such as accelerated keratinocyte differentiation, psoriasiform hyperplasia, parakeratosis, and *Malassezia* colonization-mediated inflammation. Thus, our DEM analysis presents novel molecular information underlying typical features of chronic skin disorders with respect to the miRNA expression pattern, although molecular validation of the DEMs and corresponding targetome in SD is required.

To our knowledge, this is the first study to report the DEM profiles and their potential targets in SD. Nevertheless, there are a few limitations. First, although SD is not gender-specific nor an aging disease, subjects were restricted to elderly males. This study was performed in the Veterans hospital, where the main patient group was ex-service personnel and most of them were elderly men. The results from this group may not be appropriate to apply to the general population. Nevertheless, our results provide a profound first step in elucidating the pathogenesis of SD because elderly men account for a major proportion of SD patients. Second, the number of recruited subjects was limited. Due to these limitations, further studies involving various age ranges and female subjects are required to gain further insights into this disease.

miRNA profiling has been used as a diagnostic tool for several diseases^31–34^. We evaluated miRNAs in skin biopsy samples comprising epidermal and dermal tissues. Our results suggest that the progression and status of SD pathogenesis can be understood and monitored using differential miRNA expression profiles. However, small RNA-sequencing combined with mRNA sequencing or Argonaute-crosslinking and immunoprecipitation sequencing^35,36^ may be more useful for discovering highly competent miRNA targetomes together with miRNA profiles. Additionally, state-of-the-art single-cell small RNA-sequencing methodologies^37^ can be applied to improve the understanding of differential miRNA profiles in each cell component of clinical samples in SD and many other skin diseases.

## Methods

### Patients

Five male patients with SD (mean age 74.6 years, range 72–77 years) were recruited for this study. None of the participants had been treated with immunosuppressive or antifungal agents (at least 2 weeks topical and 4 weeks systemic treatment) before volunteering. This study was approved and monitored by the Institutional Review Board (IRB) of the Veterans Health Service Medical Center, Republic of Korea (IRB no.: 2018-08-028) and conducted in compliance with the Declaration of Helsinki. Written informed consent was obtained from all study subjects.

### Clinical sample biopsies and tissue handling

Two 2-mm skin punch biopsy samples were collected from the patients, one from the lesional area and one from the non-lesional region of the scalp. The biopsy sites were selected carefully using dermoscopy. The skin biopsies were immediately placed in RNA*later*™ Stabilization Solution (AM7021, Thermo Fisher Scientific, Waltham, MA, USA) and stored at − 80 °C until RNA extraction.

### RNA extraction and microarray

Total RNA from lesional and non-lesional skin was extracted using TRIzol™ Reagent (15596026, Thermo Fisher Scientific) according to the manufacturer’s instructions. RNA purity and integrity were evaluated using the OD 260/280 ratio and analyzed using an Agilent 2100 Bioanalyzer (Agilent Technologies, Santa Clara, CA, USA) with an RNA integrity number value greater than or equal to 8. As RNA contaminated with DNA results in underestimation of the amount of RNA used, we treated the samples with DNase using an Agilent RNA 6000 Nano kit (5067-1511, Agilent Technologies). We checked the small RNA integrity using an Agilent Small RNA Kit (5067-1548, Agilent Technologies) and small RNA quantity using a Quantus Fluorometer with a Quant-iT microRNA Assay Kit (Q32882, Thermo Fisher Scientific). miRNA expression was analyzed at Macrogen, Inc. (Seoul, Korea) by performing a microarray on an Affymetrix GeneChip^®^ miRNA 4.0 Array (*Homo sapiens*). Briefly, 1 µg total RNA per sample was poly(A)-tailed, ligated, and biotinylated using a FlashTag™ Biotin Labeling Kit (Genisphere, Hatfield, PA, USA). The labeled RNA was quantified, fractionated, and hybridized into the miRNA microarray according to the manufacturer’s instructions. The labeled RNA was heated to 99 °C for 5 min and then to 45 °C for 5 min. RNA-array hybridization was performed with agitation at 60 rotations per minute for 16 h at 48 °C on an Affymetrix^®^ 450 Fluidics Station (Santa Clara, CA, USA). The chips were washed and stained using a GeneChip Fluidics Station 450 (Affymetrix) and then scanned with an Affymetrix GCS 3000 scanner (Affymetrix). Signal values were computed using the Affymetrix^®^ GeneChip™ Command Console software.

### Microarray data preprocessing

The CEL files were extracted automatically in Affymetrix data extraction protocol using GeneChip Command Console Software (AGCC) provided by the manufacturer and summarized in a tab-separated table using apt-probeset-summarize in Analysis Power Tools, with options “-a rma” for robust multi‐array average^38^ and “-a dabg” for detection above background (DABG) signal correction. Probeset information was identified using the miRNA-4_0-st-v1.pgf and miRNA-4_0.clf files, and background probes were specified for DABG signal correction using the miRNA-4_0-st-v1.bgp file. Each probe set was annotated using the NetAffx Annotation file (miRNA-4_0-st-v1.annotations.20160922.csv). pgf, .clf, .bgp, and NetAffx Annotation files were downloaded from the Affymetrix website (http://www.affymetrix.com/site/include/byproduct.affx?product=miRNAGalaxy). Data were filtered by probeset annotated with “miRNA” and “*Homo sapiens*” and by DABG P-value (*P* < 0.05). The entire data in a probeset were removed when the remaining biological replicates had fewer than three lesional and non-lesional pairs. Data annotation and filtering were conducted using in-house Python3 scripts.

### Analysis of DEMs

Comparative analysis was conducted between the N and L group samples based on the FC (*log*_*2*_*|FC|* > 0.5) and paired *t*-tests (*P* < 0.05). The *P*-value was calculated using the Pingouin package^39^. Hierarchical clustering was performed using complete linkage and Euclidean distance to measure the similarity of the DEMs. Correlation matrix analysis and PCA were performed using SciPy and Scikit-learn, respectively. Visualization for DEM analysis was performed using in-house Python3 scripts.

### qRT-PCR of mature miRNAs

The level of mature miRNAs was measured with either TaqMan^®^ MicroRNA Assays (Thermo Fisher Scientific, #001973 for U6 snRNA, #000580 for hsa-miR-20a-5p, #002260 for hsa-miR-342-3p, #000452 for hsa-miR-127-3p, #002299 for hsa-miR-191-5p, and #000442 for hsa-miR-106b-5p) or TaqMan^®^ Advanced MicroRNA Assays (Thermo Fisher Scientific, #480412_mir for hsa-miR-6824-5p, #480426_mir for hsa-miR-6831-5p, and #480541_mir for hsa-miR-7107-5p) following the manufacturer's instructions. U6 snRNA was used as the internal control for the calculation of the relative abundance of each miRNA. The lative intensity of miRNAs in L groups was further normalized by the mean intensity of miRNAs in N groups.

### miRNA target identification

Target mRNAs of each miRNA were identified either experimentally or through computational prediction of miRNA–mRNA interactions. To identify experimentally validated interactions, we used information from Tarbase^40^, miRecords^41^, and Ingenuity Knowledge Base of QIAGEN IPA^42^. For computationally predicted interactions, we used TargetScan (release version 8.0, September 2021)^43,44^. To select confident targets, the experimentally unvalidated but predicted target mRNAs were discarded when their cumulative weighted context++ scores were higher than − 0.4. These analyses were performed using IPA.

### Enrichment analysis of functional terms

Functional enrichment analysis was conducted to summarize the functions of the DEM target mRNAs. Statistical enrichment analysis was performed using g:GOSt in gProfiler^45^. For the statistical domain scope, target mRNAs of each miRNA were used as the custom background. For multiple testing corrections, the g:SCS algorithm was applied^46^. We used KEGG^47,48^, WP^49^, and REACTOME^50–52^.

### Pathway analysis

The network for several DEMs and their targets was generated using IPA (release version July 2022)^42^. The directions of FC in mRNAs were simply predicted as the additive inverse of those of miRNAs. The inverse FC of the most highly expressed miRNA was considered for genes targeted by more than one miRNA. The QIAGEN IPA z-score of each pathway was calculated using the following equation:

$$ {\text{QIAGEN}}\;{\text{IPA}}\;{\text{z - score}} = \frac{{N_{matches} - N_{mismatches} }}{{\sqrt {N_{matches} + N_{mismatches} } }} $$

where *N*_*matches*_ is the number of correct matches and *N*_*mismatches*_ is the number of incorrect matches between the FC directions of the query and reference entities in the IPA.

## Supplementary Information

Supplementary Table 1.

Supplementary Table 2.

Supplementary Table 3.

Supplementary Figures.

## Data Availability

All data generated or analyzed during the study are included in this published article and its supplementary information files.
