# Supplementary material for: Differential microRNA profiles in elderly males with seborrheic dermatitis
Source: Sci Rep. 2022 Dec 8;12:21241. doi: 10.1038/s41598-022-24383-3 (PMC9732001; doi:10.1038/s41598-022-24383-3)
Supplement: Supplementary file 4 — Supplementary Figures. [file 41598_2022_24383_MOESM4_ESM.pdf]

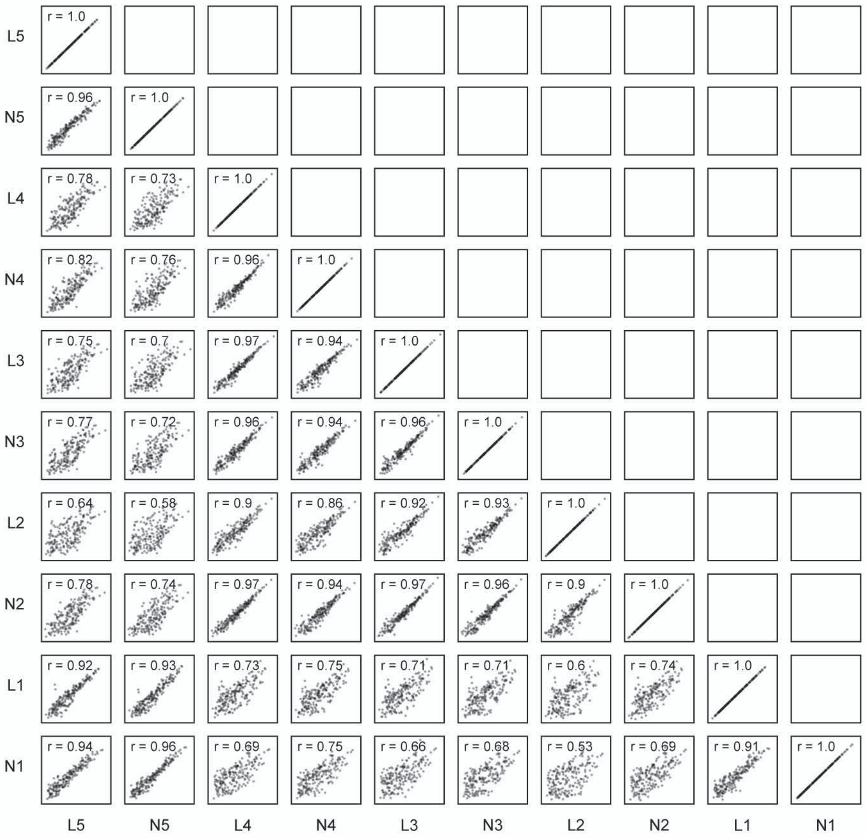

Supplementary Figure 1. Intersample correlations of normalized intensities.

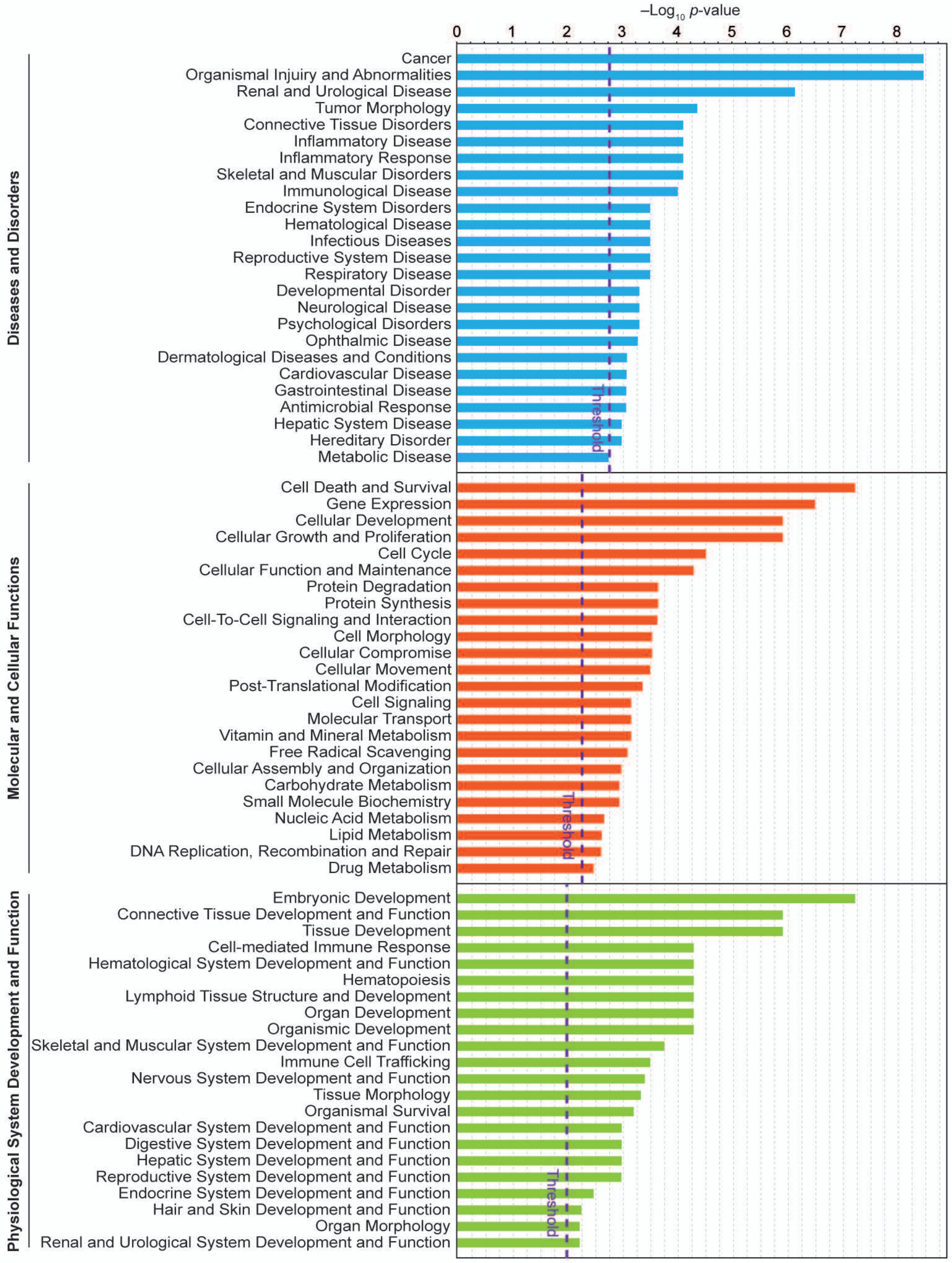

Supplementary Figure 2. Pathway analysis regarding diseases and functions using the IPA for SD-regulated target mRNAs of DEMs. Each chatergory represents as (1) disease and disorders (blue coloered bars), molecular and cellular functions (orange coloered bars), and physiological system development and function (green colored bars). The P-value was calculated using right-tailed Fisher's exact test. The threshold of –log(P-value) in the bar chart are indicated.
